# The Sankara Nethralaya Tamil Nadu Essilor Myopia (STEM) Study—Defining a Threshold for Non-Cycloplegic Myopia Prevalence in Children

**DOI:** 10.3390/jcm10061215

**Published:** 2021-03-15

**Authors:** Aparna Gopalakrishnan, Jameel Rizwana Hussaindeen, Viswanathan Sivaraman, Meenakshi Swaminathan, Yee Ling Wong, James Andrew Armitage, Alex Gentle, Simon Backhouse

**Affiliations:** 1 Myopia Clinic, Sankara Nethralaya, Unit of Medical Research Foundation, Chennai 600 006, India; rizwanaopto@gmail.com (J.R.H.); viswa@snmail.org (V.S.); drms@snmail.org (M.S.); 2 School of Medicine, Faculty of Health, Deakin University, Waurn Ponds, VIC 3216, Australia; j.armitage@deakin.edu.au (J.A.A.); alex.gentle@deakin.edu.au (A.G.); simon.backhouse@deakin.edu.au (S.B.); 3 R&D AMERA, Essilor International Singapore, Singapore 339346, Singapore; yeeling.wong@essilor.com.sg

**Keywords:** cycloplegic refraction, non-cycloplegic refraction, myopia, refractive error, open field auto refraction, prevalence

## Abstract

The aim of this study was to investigate the agreement between cycloplegic and non-cycloplegic autorefraction with an open-field auto refractor in a school vision screening set up, and to define a threshold for myopia that agrees with the standard cycloplegic refraction threshold. The study was conducted as part of the Sankara Nethralaya Tamil Nadu Essilor Myopia (STEM) study, which investigated the prevalence, incidence, and risk factors for myopia among children in South India. Children from two schools aged 5 to 15 years, with no ocular abnormalities and whose parents gave informed consent for cycloplegic refraction were included in the study. All the children underwent visual acuity assessment (Pocket Vision Screener, Elite school of Optometry, India), followed by non-cycloplegic and cycloplegic (1% tropicamide) open-field autorefraction (Grand Seiko, WAM-5500). A total of 387 children were included in the study, of whom 201 were boys. The mean (SD) age of the children was 12.2 (±2.1) years. Overall, the mean difference between cycloplegic and non-cycloplegic spherical equivalent (SE) open-field autorefraction measures was 0.34 D (limits of agreement (LOA), 1.06 D to −0.38 D). For myopes, the mean difference between cycloplegic and non-cycloplegic SE was 0.13 D (LOA, 0.63D to −0.36D). The prevalence of myopia was 12% (95% CI, 8% to 15%) using the threshold of cycloplegic SE ≤ −0.50 D, and was 14% (95% CI, 11% to 17%) with SE ≤ −0.50 D using non-cycloplegic refraction. When myopia was defined as SE of ≤−0.75 D under non-cycloplegic conditions, there was no difference between cycloplegic and non-cycloplegic open-field autorefraction prevalence estimates (12%; 95% CI, 8% to 15%; *p* = 1.00). Overall, non-cycloplegic refraction underestimates hyperopia and overestimates myopia; but for subjects with myopia, this difference is minimal and not clinically significant. A threshold of SE ≤ −0.75 D agrees well for the estimation of myopia prevalence among children when using non-cycloplegic refraction and is comparable with the standard definition of cycloplegic myopic refraction of SE ≤ −0.50 D.

## 1. Introduction

Uncorrected refractive error remains the second most common cause of visual impairment next to cataract among children [1,2]. Uncorrected refractive error among school children can negatively impact vision-related quality of life [2], and thus early detection and appropriate management is of great importance. This is particularly important as there has been a steady rise in the prevalence of myopia, and it is projected that 50% of the world’s population would be myopic by 2050 [3]. Furthermore, high myopia is associated with various vision threatening ocular morbidities such as retinal detachment, glaucoma, and myopic maculopathy [4].

There is also a substantial increase in the prevalence of myopia from 4% [5,6,7] to 13%, [8] which has been observed over the last two decades in India. Hence, there is a need for comprehensive school vision screening programs to identify uncorrected refractive errors, create awareness and implement public health policies. Generally, estimates based on non-cycloplegic refraction tend to overestimate myopia and underestimate hyperopia and their validity to diagnose and assess the prevalence of refractive errors has been questioned [9,10,11]. The main reason behind this is the influence of proximal accommodation and ‘instrument myopia’ in closed-field autorefractors [12]. Open-field autorefractors aim to eliminate this proximal accommodation cue by providing a binocular field of view of a distant target, and accommodation control [13].

Cycloplegia is the gold standard to estimate refractive errors among children [14,15], but the use of cycloplegia is restricted and can be done only in the presence of an ophthalmologist in India [16]. This restriction, the limited resources of both ophthalmologists and optometrists, and the disparity that exists between the available resources and the demand [16,17], present barriers when undertaking large refractive error screening studies in India. Another factor is the difficulty obtaining consent from parents for cycloplegia of their child, possibly due to the side effects associated with cycloplegics. Cyclopentolate is the preferred choice of drug for cycloplegic refractions but this drug is known to have both delayed onset and recovery [18]. Tropicamide is also a preferred cycloplegic agent and unlike cyclopentolate, this drug has rapid onset and quick recovery [18]. The International Myopia Institute (IMI) has recommended the use of 1% tropicamide as the cycloplegic agent in studies related to myopia [19].

In addition to the measurement technique used, prevalence estimates of refractive errors are also influenced by the threshold set. The definition of myopia varies across different studies, ranging from a cut off of <−0.25 diopters (D) [20] to ≤−1.00 D [21,22]. The recent consensus is that the definition of myopia should be based on cycloplegic refraction, with a proposed cut off of spherical equivalent (SE) ≤−0.50 D [23]. The IMI recommends defining myopia with a higher cut off (SE ≤ −0.75 D or SE ≤ −1.00 D) in the absence of cycloplegia [23], but to date, no study has examined whether proximal accommodation has a significant effect when determining the prevalence of myopia (with a threshold of SE ≤ −0.75 D) when open-field autorefraction is used.

Therefore, the aim of the present study was to compare cycloplegic and non-cycloplegic open-field autorefraction measures in a school screening program in South India, and to empirically define a threshold for myopia prevalence estimates under non-cycloplegic conditions that closely align with cycloplegic estimates.

## 2. Materials and Methods

The study was a part of a comprehensive school vision screening program, the Sankara Nethralaya Tamil Nadu Essilor Myopia (STEM) study, which aimed at identifying the prevalence and risk factors for myopia among 5 to 15-year-old children in Southern India with an estimated sample size of 12,414. A sub-cohort of the STEM study from the first two schools that underwent cycloplegic refraction formed the sample for the present study. The study protocol followed the tenets of the Declaration of Helsinki. The study was approved by the Institutional Review Board (Ethics Committee), Vision Research Foundation, Sankara Nethralaya (Study 730-2018-P), and by the Deakin University Human Research Ethics Committee (DUHREC 2020-213). Prior to the screening, the investigators approached the school management and explained the process of school vision screening. Consent was sought for the school vision screening program from the school administration. Written informed consent was obtained from the parents of all children for vision screening and cycloplegic refraction prior to commencing data collection. In addition, an oral assent was also sought from the children prior to the procedures. Data from two schools that had refraction measurements before and after cycloplegia formed the sample for this study.

### 2.1. Vision Screening and Non-Cycloplegic Autorefraction

All the children underwent monocular visual acuity assessment with a Pocket Vision Screener (PVS, Elite School of Optometry, Chennai, India) [24], which had three rows of 6/9 size letters. Children with ocular abnormalities other than refractive errors, based on the vision check, refraction, and torch light assessment, were excluded. Children then underwent non-cycloplegic autorefraction using an open-field autorefractor (Grand Seiko WAM-5500, Shigiya Machinery Works Ltd., Fukuyama, Japan). Children were asked to fixate on a non-accommodative target at a distance of 6 m, and an average of five readings (as calculated by the autorefractor) was taken as each subject’s refractive error measurement. A single examiner carried out all the open-field autorefractor measurements.

### 2.2. Cycloplegic Autorefraction

Tropicamide 1% was used to induce cycloplegia following the recommendation of the Myopia Epidemiological Study Protocol proposed by the IMI [19]. Two drops of 1% tropicamide were instilled in each eye with an interval of 5 min. Cycloplegic open-field autorefraction was performed 35 min after the instillation of the last drop. Before performing cycloplegic autorefraction, the pupillary reaction to light and near visual acuity were measured to ascertain the status of cycloplegia. An additional drop of 1% tropicamide was instilled if the child’s pupil still reacted to light or if the child was able to read the N6 near target. Cycloplegic refraction was performed during the weekend so that it did not interfere with school activities. None of the children developed any side effects to cycloplegia.

### 2.3. Definition of Cycloplegic Refractive Error

Myopia was defined as a cycloplegic SE refraction of ≤−0.50 D [23] and hyperopia was defined as SE of +2.00 D or greater [25]. Emmetropia was defined as SE ranging between 1.99 and −0.49 D. Astigmatism was defined as cylindrical correction of ≥1.00 D (negative cylinder form).

### 2.4. Statistical Analysis

Data analysis was done using SPSS software package version 20 (SPSS Inc., Chicago, IL, USA) and MedCalc Statistical Software version 19.2. 6 (MedCalc Software bv, Ostend, Belgium https://www.medcalc.org; accessed on 27 January 2021). Refractive error was converted to power vector form of M, J0 and J45 [26] for the purpose of analysis. Paired *t*-tests were used to compare refraction measurements between the two eyes. Prevalence of myopia was expressed as percentage with 95% confidence intervals. Agreement between cycloplegic and non-cycloplegic open field refraction estimates was determined using the method of Bland and Altman [27]. Sensitivity analysis was done for the three non-cycloplegic thresholds −0.50 D, −0.75 D, and −1.00 D against the cycloplegic threshold of −0.50 D for myopia. A Z test for proportions was used to compare the proportion of myopes detected using cycloplegic and non-cycloplegic thresholds of myopia.

## 3. Results

Parental consent to participate in the vision screening was obtained from 785 children from the two schools, of which only 387 (49%) children had consent for cycloplegic refraction from their parents. A total of 387 children who underwent both cycloplegic and non-cycloplegic autorefraction were included in the study analysis. The mean (SD) age of the children was 12.2 (±2.1) years (range: 5 to 15 years) and there was a balance of gender (186 (48%) girls and 201 (52%) boys). There was no statistically significant difference in refraction measurements between the two eyes for either cycloplegic (paired *t*-test; *p* = 0.08) or non-cycloplegic (paired *t*-test; *p* = 0.20) SE refraction components; therefore, only the right eye measurements were taken for analysis. Myopia was the prominent refractive error constituting 12% (*n* = 45) of the study population and astigmatism was present among 8% (*n* = 33). Hyperopia accounted for 2% (*n* = 6) of the total refractive errors. The majority of participants (81%; *n* = 314) had emmetropia. The distribution of refractive errors in the right eye are shown in Table 1.

### 3.1. Agreement between Cycloplegic and Non-Cycloplegic Refraction in the Entire Sample

**Figure 1:**
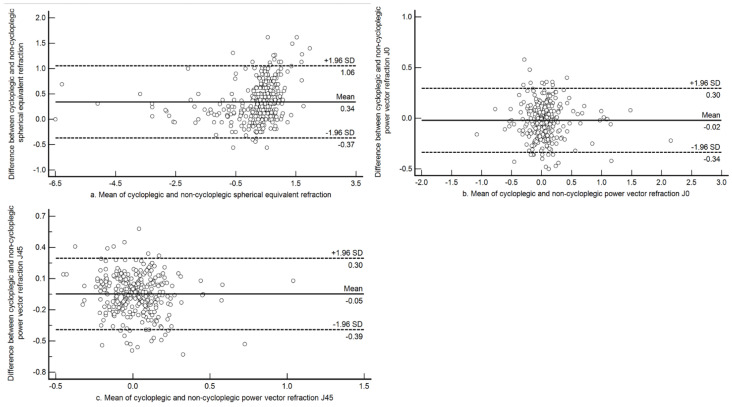
Bland-Altman plot assessing agreement between cycloplegic and non-cycloplegic refraction estimates for spherical equivalent refraction, J0 and J45 among all the children in the sample. (**a**) Agreement between cycloplegic and non-cycloplegic refraction for M; (**b**) agreement between cycloplegic and non-cycloplegic refraction for J0; (**c**) agreement between cycloplegic and non-cycloplegic refraction for J45. The central solid line represents the mean difference between the two measurements, and the broken lines above and below represents the 95% limits of agreement.

Bland and Altman plots between cycloplegic and non-cycloplegic open field auto refraction for the power vector components M, J0, and J45 in the overall sample are shown in Figure 1. The mean difference between cycloplegic and non-cycloplegic mean sphere refractions for the entire sample was +0.34 D (±0.36 D), although the 95% limits of agreement (LOA) were wider (−0.37 D to +1.05 D). Underestimation of hyperopia increased as the refraction shifted towards more plus. For the astigmatic components, the mean difference was close to zero and the limits were narrower. The mean difference was −0.02 D (±0.16 D; 95% LOA −0.34 D to +0.30 D) for J0 and −0.05 D (±0.18 D; 95% LOA −0.39 D to +0.30D) for J45.

### 3.2. Agreement between Cycloplegic and Non-Cycloplegic Refraction among Myopes

**Figure 2:**
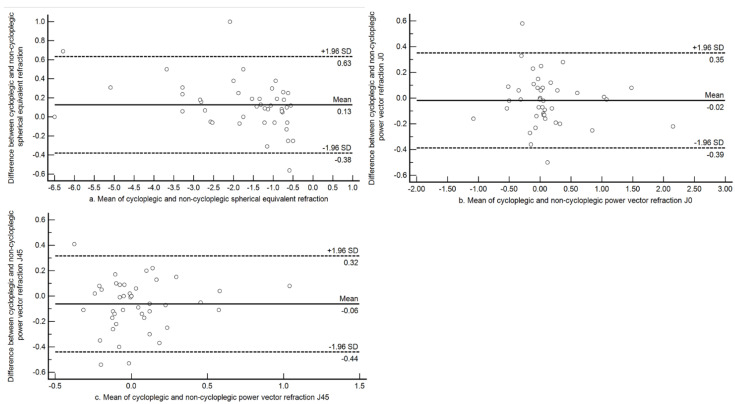
Bland-Altman plot assessing agreement between cycloplegic and non-cycloplegic refraction estimates for spherical equivalent refraction, J0 and J45 among subjects with myopia. (**a**) Agreement between cycloplegic and non-cycloplegic refraction for M; (**b**) agreement between cycloplegic and non-cycloplegic refraction for J0; (**c**) agreement between cycloplegic and non-cycloplegic refraction for J45. The central solid line represents the mean difference between the two measurements and the broken lines above and below represents the 95% Limits of Agreement.

The agreement between the two methods among myopes is shown in Figure 2. Among myopes, the mean difference was less than 0.25 D, and the 95% limits were narrower than for the whole sample (mean difference +0.13 D (±0.26 D); 95% LOA −0.38 D to +0.63 D). The astigmatic components had a mean difference close to zero with narrow limits for both J0 and J45. For J0, the mean difference was −0.02 D (±0.19 D; 95% LOA −0.39 D to +0.35 D) and for J45, the mean difference was −0.06 D (±0.18 D; 95% LOA −0.44 D to +0.32 D).

### 3.3. Agreement between Cycloplegic and Non-Cycloplegic Refraction among Hyperopes:

**Figure 3:**
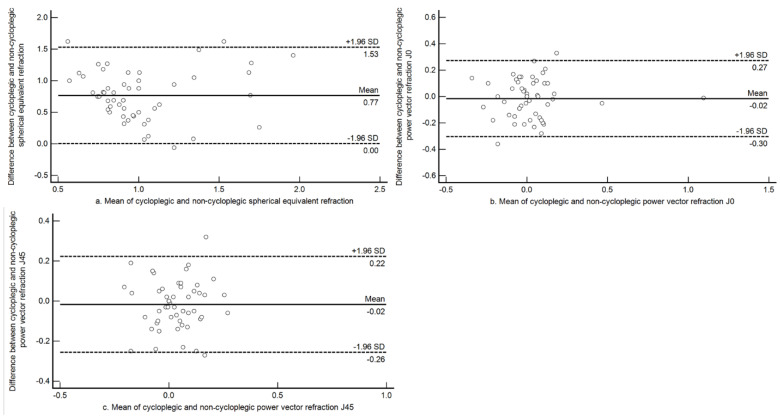
Bland-Altman plots assessing agreement between cycloplegic and non-cycloplegic refraction estimates for spherical equivalent refraction, J0 and J45 among subjects with hyperopia of greater than +1.00 D. (**a**) Agreement between cycloplegic and non-cycloplegic refraction for M; (**b**) agreement between cycloplegic and non-cycloplegic refraction for J0; (**c**) agreement between cycloplegic and non-cycloplegic refraction for J45. The central solid line represents the mean difference between the two measurements and the broken lines above and below represents the 95% Limits of Agreement.

The mean difference between cycloplegic and non-cycloplegic SE among hyperopes (*n* = 6) was +1.28 D (±0.30 D). Since there were only six children with significant hyperopia (>+2.00 D), a definition of hyperopic error of greater than +1.00 D was used to construct agreement plots. There were a total of 54 children with a refractive error >+1.00 D under cycloplegia. Bland and Altman plots between cycloplegic and non-cycloplegic open field auto refraction for the power vector components M, J0 and J45 in this group are shown in Figure 3. The mean difference among these children between cycloplegic and non-cycloplegic refraction was +0.77 D (±0.39 D) and the 95% limits of agreement (LOA) were wider (+0.01 D to +1.53 D). The astigmatic components had mean differences close to zero, with narrow limits for both J0 and J45. For J0, the mean difference was −0.02 D (±0.15 D; 95% LOA −0.30 D to +0.27 D) and for J45, the mean difference was −0.02 D (±0.12 D; 95% LOA −0.26 D to +0.22 D).

### 3.4. Sensitivity and Specificity

Sensitivity specificity and accuracy were calculated for different non-cycloplegic SE cutoffs for the presence of myopia against the standard definition of SE ≤ −0.50 D under cycloplegia (Table 2).

### 3.5. Prevalence Estimates of Myopia with Different Thresholds of Myopia (Non-Cycloplegic) Compared to Standard Cycloplegic Refraction Threshold

When myopia was defined as SE ≤ −0.50 D in both non-cycloplegic and cycloplegic refractions, the myopia prevalence was 14% (95% CI 11% to 17%) under non-cycloplegic refraction and 12% (95% CI 8% to 15%) with cycloplegic refraction. With the stricter myopia threshold of SE ≤ −0.75 D under non-cycloplegic condition, the number of false positives decreased and the prevalence estimate was 12% (95% CI 8% to 15%) under both non-cycloplegic (SE ≤ −0.75 D) and cycloplegic refraction estimates (the standard SE ≤ −0.50 D) (*p* = 1.00, two sample test of proportions).

## 4. Discussion

We found that a higher non-cycloplegic threshold (SE ≤ −0.75 D) to define myopia along with using an open-field autorefractor improved the accuracy of myopia prevalence estimates and was comparable with cycloplegic refraction. Previous studies comparing cycloplegic and non-cycloplegic refraction estimates have in general found an overestimation of myopia estimates in the absence of cycloplegia [9,10,11,12,28,29,30,31].

The difference between cycloplegic and non-cycloplegic refraction measurements varies depending on the type of instrument used to assess the refraction [32]. A study comparing retinoscopy and autorefraction found non-cycloplegic retinoscopy to have a lesser mean difference between cycloplegic and non-cycloplegic findings (−0.37 D) when compared with autorefractor measurements (−0.86 D) and concluded that non-cycloplegic retinoscopy may be a better starting point for subjective refraction in young adults [33]. Studies that used closed-field autorefractors [10,11] had a higher mean difference when compared with studies that assessed refraction using open-field autorefractors [12,30,31]. In our study, we found a mean difference of +0.34 D when refractions were compared without and with cycloplegia, which is similar to a recent study [12] of school children in Taiwan using open-field autorefraction (mean difference +0.21 D).

Among children with myopia, we found the mean difference to be +0.13 D, which we contend is well within the clinically acceptable limits of 0.25 to 0.50 D [17,31]. Moreover, the sensitivity and specificity to identify myopia was greater than 90% with the open-field assessments under the non-cycloplegia condition even with a lower threshold. Similar results were obtained in a study by Choong et al. [30], where three different autorefractors (Retinomax K plus2, Canon RK 10, and Grand Seiko WR 5100K) were compared under both non-cycloplegic and cycloplegic conditions with subjective refraction. Open field autorefractors had the highest sensitivity and specificity (0.91 and 0.98) under non-cycloplegic conditions. In the current study we found a similar sensitivity and specificity (0.96 and 0.97).

Few studies have tried to improve the accuracy of prevalence estimates and refraction measurements with alternative methods other than cycloplegia, however the use of fogging lenses over open-field autorefraction was suggested as an alternative to cycloplegia in a study by Queirós et al. [34] especially in a community set up. In the Sydney Myopia Study, setting an uncorrected visual acuity (VA) cutoff of 6/9.5 or less as the threshold to identify refractive errors, sensitivity, and specificity for identifying myopia (≤−1.00D) was 97% [35] and the authors suggested that using unaided visual acuity as the threshold, prevalence of myopia can be estimated with reasonable accuracy. In a study by He et al. [36] combining axial length to corneal curvature ratio with uncorrected VA was a better predictor of myopia prevalence than uncorrected VA alone.

In another study among Taiwanese children, when uncorrected visual acuity was combined with non-cycloplegic cylindrical correction, the sensitivity and specificity to identify significant refractive errors improved in a vision screening set up [37]. Sankaridurg et al. [38] tried to predict refractive status based on non-cycloplegic refraction and found that the addition of visual acuity, or axial length to corneal curvature to the non-cycloplegic refraction, did not improve the prediction of refractive errors. They found that a model combining visual acuity and non-cycloplegic refraction improved the predictive accuracy of myopia (myopia defined as SE ≤ −0.75 D under cycloplegia) among children, but that this model was poor in predicting emmetropia and hyperopia.

We found a threshold of SE ≤− 0.75 D as measured by non-cycloplegic open-field autorefraction to be in agreement with the prevalence of myopia compared with a cycloplegic threshold refraction of SE ≤ −0.50 D (*p* = 1.00). Notably, this threshold is based on open-field autorefractor measurements and may not be generalizable to other methods of refraction measurements (e.g., closed-field autorefraction).

Additionally, we found that the sensitivity for hyperopia was poor as the threshold for hyperopia increased. Hence, the recommendation in a community set-up would be to refer a child for cycloplegic refraction to rule out latent hyperopia and other binocular vision anomalies, if asthenopic symptoms are present along with mild hyperopic error under non-cycloplegic conditions. However, in general, in community-based vision screening programs the prevalence of hyperopia among Indian children has been predominantly shown to be low and has remained constant [39,40,41]. The prevalence of myopia is the primary visual concern within school children [42], and adequate estimation of the prevalence is important in order to understand its impact on the country’s economic burden and in laying out public health policies.

In our study, 50% of the parents did not provide consent for cycloplegic refraction at the school premises. Alternative options for determining the prevalence of myopia in school screening projects are therefore required to mitigate this difficulty in obtaining parental consent for cycloplegia. A post-hoc power analysis of the sample size in the present study with a primary outcome measure of difference in SE estimates between cycloplegic and non-cycloplegic open field autorefraction results showed a power of 100% and an effect size of 0.94, suggesting our modified refraction threshold approach is appropriate for prevalence estimates in school screening settings.

Another important consideration is the prescription of spectacles for refractive correction by referring the children for cycloplegic refraction to the base hospital in a community screening set up. The attendance rate at the hospital for follow-up cycloplegic refraction is also poor, and there is a poor compliance with referrals [16,43]. These reasons make it essential to look for other solutions to address the problem of cycloplegic refraction in the community. In that context, open-field autorefraction is a valid and efficient tool for measuring refraction among school children under non-cycloplegic conditions, assuming that an appropriate definition of myopia is used.

To conclude, cycloplegic refraction is still necessary when estimating the prevalence of hyperopia, especially in populations where the known prevalence of hyperopia is high. Based on the findings from our study among Indian children aged 5 to 15 years in a community set up, a non-cycloplegic threshold of SE ≤ −0.75 D provides an accurate prevalence estimate of myopia.

## Figures and Tables

**Table 1:** The number of children with different cycloplegic refractive errors, the mean refractive error in each group, and the range of refraction in the right eye.

State of Refraction (n)	Mean (SD)	Range
Myopia (45)	−1.69 D (1.38 D)	−0.50 D to −6.50 D
Hyperopia (6)	+2.30 D (0.21 D)	+2.08 D to +2.66 D
Astigmatism (33)	−1.63 D (0.75 D)	−1.00 D to −4.17 D
Emmetropia (314)	+0.31 D (0.32 D)	−0.49 D to +1.99 D

**Table 2:** Sensitivity specificity and accuracy for different myopia thresholds with non-cycloplegic open-field autorefraction against gold standard cycloplegic refraction.

Myopia Threshold for Non-Cycloplegic SE	Sensitivity	Specificity	Accuracy
≤−0.50 D	96	97	97
≤−0.75 D	84	98	96
≤−1.00 D	64	99	95

## Data Availability

Data is available as Supplementary File.

## Supplementary Materials

Supplementary materials are available online at https://www.mdpi.com/2077-0383/10/6/1215/s1.
